# Proceedings of the Association for the Study of Comparative Psychology of McGill University, Montreal

**Published:** 1892-12

**Authors:** 


### SOCIETY PROCEEDINGS.

### PROCEEDINGS FOR 1891-'92 OF THE ASSOCIATION FOR THE STUDY OF COMPARATIVE PSYCHOLOGY IN CONNECTION WITH THE FACULTY OF COMPARATIVE MEDICINE AND VETERINARY SCIENCE OF McGILL UNIVERSITY, MONTREAL.

At the annual meeting held in October officers were elected, reports read and the inaugural address of the year proposed by Dr. Parker, of Haverhill, Mass. a recent graduate, was read by the Secretary. This address was much appreciated, published and distributed to graduates of this Faculty.

The officers for the year were: Hon. Pres., Dr. D. McEachran; Pres., Dr. Wesley Mills; 1st Vice-Pres., Dr. M. C. Baker; 2nd Vice-Pres., Wm. Ramsay; Sec. and Treas., Mr. S. Moffatt.

The attendance throughout the session at meetings was good and diplomas of honorary fellowship were given to those who had complied with the conditions, which were that a satisfactory thesis must be submitted.

The Association has laid the foundations of a library to which it is the intention to add annually works bearing on the subject of animal intelligence.

On Nov. 4th, papers were read by the following members: D. L. Bolger, Animal Appreciation; R. E. Dyer, Psychic Phenomena in the Lower Animals; G. E. Gangloff. The President, Prof. Wesley Mills, occupied the Chair, and the subjects treated were discussed at length.

Nov. 23rd, President in the chair. The executive reported that Dr. Parker's inaugural address had been kindly published by the *Montreal Gazette*, and that reprints had been secured and would be distributed to members for circulation. It was voted to donate twenty-five copies to the Montreal branch of the Society for the Prevention of Cruelty to Animals, a society that was in sympathy with the work of this organization and which was accomplishing much good.

Mr. Hadley read a paper in which he compared the intelligence of man and that of the lower animals. He related several instances of intelligence in the donkey and in the cat, which seemed to be much above what we are accustomed to attribute to these animals, notably in the case of an ass that delivered milk on his own account when his master was too ill to accompany him.

The president criticized this and the next paper unfavorably, inasmuch as they were not in keeping with the principles that had been laid down when the society was founded, to the effect that the facts by which views were substantiated should have come under the writer's own observation in the main at least.

Mr. Lofgren wrote upon "Psychology in Relation to Veterinary Science.' While this paper was excellent in its way, it lacked that individuality which must be indicative of independent thought on the part of the writer to insure the end in view.

The president held strongly that the object of the association is to advance the study of animal intelligence, not merely to echo the opinions of others.

Mr. Lee read a paper on "Memory etc., in our Domestic Animals," illustrating his views entirely from observations of his own. The case of a mare that had to be cast on account of restlessness under examination was instanced. Months afterwards this mare recognized the voice of the veterinarian when she was stopped near him on the road and manifested displeasure. He also referred to a small yellow mongrel that was kept in a stable with a racehorse that belonged to his master. When the horse won, the dog, which took up his stand at the finishing post, manifested delight, but the reverse was the case when his friend was not first. Mr. Lee's paper was excellent in tone and was calculated to advance the study of comparative psychology.

Oct. 14th. The President in the chair. A paper was read by Mr. Jos. Moffatt bearing on the question of the extent to which it is possible to become acquainted with the true nature of the mental processes of animals. Reference was made to the author's experience with mules, and from this he concluded that mules were, when well treated, more easily broken and more intelligent, as well as more affectionate to each other, than horses. Instances were mentioned of evident pining from the separation of mules that had been accustomed to work together.

Another member of the society thought that female mules were decidedly more intelligent than the males.

Mr. Moffatt pointed out that some individuals of different species of animals seemed to show downright perversity of disposition.

This was followed by a paper by Mr. McIntyre in a somewhat similar strain, in which he also discussed the same main question. Some individuals of the same species seemed to be very intelligent, and others very stupid. As an instance of the former he mentioned the case of a collie, a year and a half old, that separated two cocks that were fighting and drove his master's home and into the shed in which he had been shut up, and stood guarding the door. As this was repeated when the bird was intentionally let out, it was a plain case of unusual intelligence. He related the case of a horse that moved away from where he was left standing, and instead of taking the road across a bridge towards home, took to the river and attempted to swim with a buggy behind him. This was the way the road led in winter across the ice. It was considered an act of stupidity by the writer, but some members thought it capable of a different interpretation. Possibly the horse, in a sort of absent-minded way, took the accustomed winter road, and when he found himself in the water endeavored to reach the other side, not having by experience previously learned that this was impossible, but, on the contrary, that it was readily done in the winter. Men sometimes acted in a similar way; moreover, the road by the water was rather shorter.

Mr. Lamb, in a paper of a different tone, furnished evidence of the intelligence of the donkey and the goat, more especially by apt cases.

The president thought that the exact interpretation of the acts of the lower animals must ever remain difficult, but it was not impossible; at least we could get some insight into their minds. Most thoughtful people had their periods of scepticism preceding those of settled conviction, and as a rule, a mind incapable of scepticism was also incapable of high philosophical development. The object of the society was not, as he had often said, to act the special pleader for the lower animals, but to simply ascertain the truth. He believed that only those with freedom from preconceived notions and an earnest desire to know the truth would ever arrive at sound conclusions regarding the character of animal intelligence or any nature's problems. He commended the thoughtfulness of the papers read.

Jan. 19th. The President in the chair. Three papers were read. In the first, by Mr. McDougall, instances were cited and carefully analyzed to show that the acts of different animals were based on mental processes akin to our own. These related to a horse that had thrown his rider, when taken out at night, the roads being bad, and by his subsequent actions, shown that he was solicitous for his welfare; a dog that showed unusual concern when his master was fatally ill ; and a monkey that brought the cap of the ship's carpenter, to whom the animal belonged, after a fall that rendered him unconscious, on deck to one of the officers.

Mr. Pote read a paper entitled "Notes and Inferences on Comparative Psychology." It showed that the writer had carefully considered the ways of the lower animals and subjected their psychic processes to analysis, and with considerable success. Such points as the resemblance of the brains of the lower animals and that of man ; the language by which they communicate with each other ; their feelings of attachment ; their jealousies, rivalries, etc., were all alluded to and illustrated by example. It was said that the celebrated Sunol could not rest well if the attendant did not sleep in his usual place near her ; and an instance of a blind horse that had accidently walked off a bank into a river being led out by the halter by his companion horse, was cited in connection with the above in evidence of sympathy existing between the horse and man and between one horse and another.

Mr. J. Plaskett, in a well written and well read paper, discussed the scope of comparative psychology, its importance, both scientifically and practically, and supported his views by several well chosen examples. After some discussion on the papers of the evening, the president suggested several lines of research and advised all to keep a little book in which observations and reflections on the subject might be recorded, so that they might not be lost.

Feb. 4th. The President in the chair. A thoughtful paper was read by Mr. Ramsay, entitled "Observations of An Observer." The language of animals, especially of dogs and horses, was considered. A case of a crow that could repeat certain phrases was instanced. He mentioned the case of a horse, twenty-two years old, that would let down the fence bars, but not when watched. In one instance a Scotch collie restrained a little girl about to pass near a dangerous piece of machinery, apparently with a sense of the risk. A certain setter had refused to hunt and ran out of sight, though possessed of much ability, after his master had punished him for not offering himself readily for the trip when he was called. This seemed to be a sort of revenge Mr. Ramsay expressed the opinion that balkiness in horses resulted from bad management.

Mr. Robb discussed in a paper several questions bearing on comparative psychology as reason, instinct, language, etc. He differed from Rarey, the famous horse trainer, in his *dictum* that his success was due to the lack of intelligence on the part of the horse relatively to man, for in his large experience as a trainer Mr. Robb had found that the most intelligent horses were the most readily taught and governed.

Mr. Robertson read a paper on "Observations on the Lower Animals from a Psychological Standpoint." To be successful in the highest sense in understanding the intelligence of the lower animals we must thoroughly understand our own, and that was no easy task. Reference was made to the readiness with which each one of thirty cows would find her own place in a barn. How was this done? He thought they had a more accurate sense of distance than man, perhaps. Why did not other cows take down fences, etc., when some among them did? He thought it was lack of attention. Sheep, when sheared, were not at once recognized at sight by their young, but immediately when the voice was heard. He showed that while animals have great ability to find their way home from distant points, they also make mistakes.

The papers were then discussed. The president thought that all the writers had shown a good grasp of their subjects.

Feb. 15th. The President in the chair. A paper was read by Mr. Seale, entitled "Reasoning in the Lower Animals." The writer raised the question, How would a child differ in its development from, say, a dog, if unable to use articulate language? Pigs seemed to have a very expressive language at times, though limited. The case of a collie dog that learned both French and English so as to understand much that was said to him, was specially dwelt upon. Cows would betake themselves to the woods to give birth to the calf, and would resort to artifices to mislead those seeking for it. This was evidence of reasoning, for it was much more frequent in the older animals. The wiles of the fox when pursued by the hounds could not be explained by instinct alone.

Mr. Wells read an essay entitled "A Few Thoughts on Psychology " He maintained that the lower animals had practically the same faculties as ourselves though in their thinkings they did not rise to the heights of abstraction of which man was capable. He illustrated his views by a cocker dog, Fritz, of a wonderful memory, intelligence and apparent understanding of language This dog showed that he had the same qualities as human beings, such as pride, sensitiveness to reproach, etc. The intelligence of the horse was well illustrated by the behavior of the cow ponies on the ranges on which cattle were kept. Mr. Wells gave an admirable illustration of the intelligence of a cat. Her kittens had been removed from the barn to the house. They were taken back and she returned them. This being repeated some four or five times, she then went off about half a mile and brought a male cat, and he assisted in the carrying.

Mr. S. Moffat, the Secretary of the society, next read a paper on ' Animal Intelligence," in which he took much the same ground as the previous essayists.

Considerable discussion followed. The President advised the members to avoid the methods of the debating society and the special pleader, as such blinded the mind to the perception of truth. He suggested a more close and careful analysis of single cases than had yet been given by the readers of papers, and advised all to keep note-books in which facts and the suggestions to which they give rise might be stored.

Feb. 29th. The President, Prof. Wesley Mills, in the chair. Mr. Dunton read a paper entitled "Anecdotes of Animal Intelligence," which scarcely expressed its real nature. The subject of animal intelligence was discussed as well as illustrated by the writer. The case of a dog that would board the train on which his master was employed and no other was considered. An instance of close friendship between a dog and a cat, which seemed to survive the death of the latter, was related An amusing instance was told of a little dog, a Scotch terrier, that would chase a ball thrown, where he had to compete with a Newfoundland. He would seize the shaggy hair of the big dog and get pulled along till near the ball, when he would dash on and secure it.

Instances of intelligence in horses were also cited, among others one illustrating that the animal knew its own dinner horn, for when it sounded he would kick and bite his mate if not released.

Mr Rathbone read a paper on the intelligence of the horse. He referred to the capacity of this animal for education, as shown in most horses; among others those used by fire brigades; also to horses lifting off gates to enable them to escape from enclosures; to finding their way home from remote points; to their going to blacksmith forges of their own accord, in some instances to have a lamed foot re-shod, etc.

Mr McNaughton discussed "Manifestations of Intelligence in the Lower Animals." He had possessed a young collie that used to resort to a ruse to get a chance at the food set down for himself and another. The larger dog kept this one away from the dish, so the little one would rush out and bark in an excited way, and when his mate would go out to investigate, he would steal in by another door and eat the food. This same dog, that was far in advance of the other in general intelligence, would awaken any member of the household when told to do so, and when two slept in the one room he would make such investigation as enabled him to determine the right person, and would awaken him by licking the face. This writer also showed that in one case a horse understood language to a certain extent. The horses his family had owned avoided breaking into the grain fields that were easily in the sight, but chose those distant from the house.

After some discussion of the above, a paper published in the *Popular Science Monthly* for February, entitled "Is Man the Only Reasoner?" by Prof. James Sully, was read by Mr. Robb, at the President's suggestion.

---

### Comparative Psychology with Special Reference to the Veterinary Profession.*

By Professor M. C. Baker, D.V.S.

*Mr. President and Gentlemen:* –I must confess to feeling very considerably that I am in rather an awkward position. That the activity I have taken, or I might almost say that the inactivity I have manifested, does not entitle me to the honor of giving the opening address to the Psychological Society. I am a victim of excessive zeal, not by any means my own, but that of your President. He has the most powerfully persuasive manner of any one I know; in fact, it is even stronger than persuasive, as you soon will know. He one day said, "I wish you would give the opening address." To my surprise I consented, to immediately repent, but vainly. When Prof. Mills makes up his mind to get any one to do any thing, I have never known him to fail (this is a psychological problem which I will let you work out individually). But I felt that some explanation was required for my present position.

Now the objects of an opening address are most excellent. It has several objects: to outline the work of the coming session; to remind the members of what we have done and what we hope to do; and I may also add, what is to be gained by this: how we may profit by what we are doing. This latter is not by any means the smallest or least important object of the opening lecture.

---

* Being the inaugural address of the session of 1892–93, of the Association for the study of Comparative Psychology, in connection with the Faculty of Comparative Medicine and Veterinary Science of McGill Uuiversity.

1st. What we have been doing. Now, I believe, there is an impression abroad that this Association is a story-telling club, where one member vies with another in the collection of anecdotes. This, however, is not our object, it only comes in incidentally. The stories are the condiments for the solids. The object is to reason from what we see and hear. We use the stories to illustrate facts and theories. For example, an animal is noticed to do something out of the ordinary, common performances of the average member of his species. This act is carefully noted. Now, to the large proportion of those who witness the act, or to whom it may be related, there is in it no special significance. To one engaged in Psychological study, the case is very different. The first sees only on the surface; his fancy is tickled; he is supplied with material for an interesting anecdote, or it may be his fondness for the animal, particularly if he is the owner, is, for the time at least, increased. The latter looks deeper and strives to determine the impulse that prompted the act, whether it is born of pure instinct or if there is not something in the act that proclaims the possession of some higher trait or power.

This, then, is a very brief and imperfect outline of what the members of this society are trying to do. We are not merely collecting pleasant anecdotes, and developing our story-telling functions, but we are trying to determine to what extent the lower animals are actuated by instinct and how much of their actions are guided by thought, reason or intelligence.

It is perfectly natural, and to be expected, that we must use some sort of illustration to prove our belief in the possession, by the lower animals, of the higher power of reason or intelligence, and the only form of illustration we can make are of the manifestations of that higher power; or, in other words, we tell stories of our own, of our friends' animals, not for the sake of the story, but for what it proves.

During the years that this society has been in existence, there have been a very great number of most interesting as well as valuable illustrations presented to it at the regular meetings; many, if not the most, thoroughly authenticated; all showing that our dumb friends and fellow creatures do possess a certain amount of intelligence; that many of their acts are prompted by something beyond and higher than mere hereditary instinct; that they do not act in a merely reflex manner; and that in cases of emergency, and even in many of their every-day actions, they give evidence of the possession of reasoning powers, cannot be denied.

The object of this society is to study the manifestations of those powers, and of the higher faculties, that have been popularly supposed to be the exclusive prerogatives of man. No class of men have better opportunities for such study than the members of our profession. We are deeply interested, or should be, in the study of the lower animals, not only physically, but psychologically. Deprived as they are of the power of giving expression to their desires, needs or emotions by articulate language, no one doubts their experience of all these, but all cannot understand their mode of giving expression to them. The more carefully and thoughtfully their habits are studied, the more perfectly can we hope to understand and minister to them. Brought as we are into constant daily contact with greater or lesser numbers of many species of animals; actuated, we certainly should be, with a desire to know how we may best relieve their sufferings and minister to their comfort and well being, it will prove greatly to our advantage if we study carefully the psychological manifestations as well as their physical habits ; the two are much more closely alied than many of us may think. It is undoubtedly true that man's diseases are oftentimes seriously modified by mental disturbances. I am quite certain that such is frequently the case in horses, and very probably in other animals. I have very frequently noticed how much more difficult it is to successfully treat an animal that has recently come from a distant city or country than one that is at home ; and it is not, I am certain, merely due to the physical fatigue incidental to the journey, but largely due to a something closely allied to home-sickness. I have had patients die that I firmly believe would have recovered if they had not, at the same time been suffering from extreme mental depression as well as disease. You may ask what practical use can we make of this knowledge or belief. I answer that, as students of comparative psychology, we are furnished with a fresh stimulus to work on in this line; to more closely observe the effects of the environments upon our patients, and whenever an opportunity offers to see to it that whenever animals, possessing acuter sensibilities than common, that manifest strong affections, or are of an emotional temperament, having to be sent long distances from their original home, are accompanied by an accustomed attendant. Let them carry to their new home something of the old, and not go with only strangers to a strange home.

This is only one line in which our studies may be directed. But I think that it is not at all inadvisable to here state that we should always try, as far as we may, to give some practical illustration or application of the fruits of our researches and studies.

I wish only to occupy your attention for a short while, and during that short time I will try and venture upon a few suggestions that may be of use to you in stimulating and directing your workings in connection with this society, and also to try and point out some of the possibilities of practically applying, for the benefit of your fellow-creatures, the results of your researches.

It is, first, essential to acquire the habit of close observation. And I can assure you that there is no habit that will be of more service to you through life than this. Learn to see, and note what you see. Carelessness is, unfortunately, far too common with us all ; we are very apt to think that what we may see is of no importance, that it can be of no interest or use to us, and before we realize the fact we are careless observesr, do not see one-half of what there is to see, and seeing, do not perceive. We can never tell how important an incident may prove, how much we may learn from it, what bearing it may have upon our whole life even, until we have watched and studied it to its conclusion. In this particuiar line of study, as well as in every other, we can always find plenty of material for our use, if we will only be always ready to avail ourselves of it. The world is full of animals. There are always some manifesting their various traits and characteristics before us, and we, if we are in earnest in our search after truth—and the truly earnest scientific man is always looking for the truth—can always find a book, from which to learn, before us, if we will only look, look steadily and deeply.

Every animal that passes before your notice should be made an object of careful study. They have not the power of speech, but they have an immense variety of ways whereby they give expression to feelings as forcibly as if by words.

Some are much more demonstrative than others, their feelings are more easily excited, and exercising less self-restraint, they give more active evidence of their possession. Animals are very good character readers, and much prefer a sympathetic to a non-sympathetic confidant. One who is really fond of animals can learn much more from them of their reasoning powers and the degree of intelligence they may possess, than one who unconsciously perhaps, repels their advances, and, as it were, throws cold water over their first attempts to make known their wishes.

If we wish to ascertain the existence of anything in the material or psychological world, we must be ever ready to recognize all the evidences that have any bearing for or against it, that we may be fortunate enough to have brought before us, whether it is of a startling, unusual character, or only some commonplace incident of every-day occurrence.

The behavior of an animal that is, for the first time, placed in an unusual or strange position, as well as one in its every-day routine, will afford material for study. That the affections of the lower animals are strong, and even lasting, is a fact that requires no evidence of mine to prove. Who has not noticed, repeatedly, how fond a dog is of one who is habitually kind to him, or how great is his aversion towards one who neglects or ill-uses him. Instinct is strong in all animals, high or low. But they are also influenced in their affections by a higher force. Selfishness is strong; an animal soon learns from whom he can obtain the most material assistance or sustenance, who there is about him that will most indulge him in all his desires, and he also soon learns what are the means he must employ that will be most likely to gain for him their gratification. Who has not been amused with the antics of a dog that has been taught to beg—this may become a more or less reflex act in time, but it does show the feeling of a want and the knowledge that certain actions on his part will lead to his wants being relieved, and it is quite astonishing how much discrimination is manifested by the voiceless beggar.

He does not waste his time or talents long in the presence of an unsympathetic person, but to one who has heeded his requests in the past he is as importunate as any old woman who asks the price of a loaf from one who has never refused her in the past. This is a very simple instance of acquired knowledge on the part of one of the lower animals, and one that has for its stimulus a want or desire to be gratified. But at the same time it is an evidence that the animal reasons that if he only perseveres he will get what he wants, or a substitute for it at all events. The recognition of a long lost friend, that is often shown by dogs, as well as other animals, indicates that they possess memory as well as affection. On the other hand, you have often heard some one say, that dog always growls every time he sees *So-and-So*. Why is it? we ask; and if we pursue the investigation, we will find that *So-and-So*, at some time or other, has done something to offend the dog, and has not been either forgiven or forgotten.

The various tricks that animals may be taught are not all merely reflex acts; certainly not at first, however they may finally become, but are manifestations of the possession of something higher than instinct. I do not deny that the reflexes are very powerful in the lower, as well as the higher animal, but these can be extended, enlarged and modified by training and surroundings.

The members of this society are, presumably, anxious to ascertain by their own efforts, to prove by the result of their own observations, and to determine for their own and fellow-members' satisfaction, how far instinct, or hereditary reflex, acts, prompts, regulates, and governs the acts of animals, and to what extent they are influenced by education and guided by reason.

Comparative psychology is a large study; there are no limits to its possibilities; and the endless variety of means that may be adopted in its prosecution, as well as abundance of material from which to gather facts, make it a most interesting study, and I think, too, it can be made a most valuable one to us, who have to so constantly deal with animals. Take for example, the training of a colt Nearly all boys from the country have had more or less experience in this, and know to some extent what it is like. The first requisite on the part of the trainer is patience. A man must first have himself in good subjection, or he cannot hope to educate another animal to obey his will, for the first thing necessary is to convince the colt that yours is the stronger will, then teach him your pleasure and he will do it. I need not dilate here upon the importance of kindness, for that is obvious. because you wish to convince the colt that yours is the stronger will. Why, then, arouse in him any unnecessary antagonism to you? Rather do the reverse, soothe him ; convince him by your actions that it is your desire to be friendly, and that if he will permit it you will be so.

I think we can safely conclude that since we find from practical experience that by a rational exercise of kindness and firmness we educate our colt much more quickly, and at the same time more thoroughly. than by the mere exercise of force; that our kindness appeals to a higher power than mere instinct; that our reason acts upon his, the lesser yields to the greater, and our colt is thereby broken or educated. Did you ever see a vicious tail-switcher, one that seemed all the time trying to catch the rein, and, when he succeeded, hold it tight, or perhaps kick and make himself generally disagreeable? Look into the history of that horse and study him carefully. In the great majority of cases you will find that at first there was no motive in the catching of the rein, merely the ordinary act of the horse to rid himself of some real or fancied irritant that his instinct told him he could do with his tail. He accidentally catches the rein. The driver is either annoyed or frightened. From one cause or other he loses his presence of mind, and gives a vicious tug at the rein, and perhaps, I am sorry to say but too often, punishes the poor animal for what was partly an accident (entirely so so far as the horse is concerned) and partly the fault of the careless driver in allowing the rein to lie in the way of his horse's tail. What is the result of this harshness? The poor animal is frightened, perhaps annoyed, at least irritated, switches his tail in a nervous manner, catches the rein again, to have it again jerked out, and now he is surely punished. and the driver mutters to himself and partly to his dumb servitor, "I'll teach you, you brute!" He may swear a little. He tries to think he has done the right thing, but he soon finds that he has sown for himself trouble that he will soon reap. The next time the rein is caught it is not only tightly held by the poor frightened beast, but in its nervousness it may do almost anything it ought not—kick, attempt to bolt, or even lie down—to be most likely again punished. In a very short time the bad habit is formed that will take a very long time to be overcome and perhaps can never be entirely eradicated. A little gentleness and coolness on the part of the driver would have prevented all this trouble If the driver loses his head and temper, how can he wonder that the horse will lose his also? Man claims to be superior to the dumb animals in intelligence, but he oftentimes fails to prove himself so in an emergency.

One of the most trying and exasperating things a horse can do is to shy. Some are worse than others. Some only show fear at certain objects; others have no discrimination apparently, but are alarmed at any and every thing; and it is sometimes very difficult to cure them of the habit. But in the very great majority of cases you will find that harsh measures are worse than useless. Gentleness and firmness will accomplish much; whipping does harm. It is often caused by defective eyesight, an inability to judge distances; an object seems to appear to them nearer than it really is. Such cases are the very hardest to cure. The only way is, to go slow and let them learn that their driver is their friend who never asks them to face any real danger. Praise them when they overcome the feeling of fear. Let them have a good look at the object, and nine out of ten will pass it next time.

Such trifling incidents as I have just mentioned are only one or two of many that will afford us abundant opportunity of pursuing of our studies in investigating the reasoning powers of the lower animals, which will amply repay us. A man who studies animals must study himself ; he must practice self restraint if he hopes to restrain others, be they animals or men, and by so doing he not only increases his store of knowledge, but strengthens and elevates his own character.

We should at all times, in our dealings with animals, endeavor to remember that they are not mere unreasoning beasts, but that they possess feelings and affections. Their possession of a highly developed nervous organization is a proof that they suffer bodily pain and experience pleasure in a degree only slightly, if any, less than man, and many of their actions equally prove that they also possess very considerable capabilities of suffering and enjoyment mentally, if I may use this term to express my meaning ; that is, they appreciate kindness and efforts made on their behalf, and suffer from neglect and cruelty, even when no physical injury is inflicted.

I do not claim that we can make an equal of the lower animals; they will ever remain lower in the scale than man, but they deserve a consideration of their faculties and powers always in so far as it is possible and should be our constant aim to develop their higher or better nature by never ignoring its existence. We should take care that their bodies are made comfortable, for the intimate connection between the body and the mind that exists in man, must also obtain in the ower animals. Some one has said that the first step insocial reform must be good food, plenty of fresh air and soap and water. This is also the first step needed with animals—feed them well, give them plenty of fresh air to breath, and whilst soap and water may not be just the thing, cleanliness is certainly most essential. Compare the horse, that is in the fullest sense well cared for with the poorly-fed, badly housed and filthy animal; what a contrast there is in the character of the two? The difference is largely due to the surroundings; change the place and you also change their whole being. The horses that give evidence of the highest educational attainments, whose remarkable actions show such a high possibility, the performing horses of the show ring are always well kept and well cared for. I have no doubt but that their surrounding have done a great deal towards increasing their mental powers, have at least made them capable of greater progress in their development. It is also important that you give the animal credit for the possession of some reasoning power, if you hope to have a manifestation of its existence. If you use rough, harsh measures, cruel blows, and equally cruel words, you need not be disappointed if your animal fails to be anything but a perverse, unmanageable brute On the other hand, if you quietly, but firmly, make him understand what you wish him to do, and by never urging or inducing him to do anything impossible or beyond his power, praising his efforts in the right direction and not dealing too harshly with his failure to understand what you want him to do, or his unnwillingness to do it; when you think he knows what it is, you may be assured that if you will but persevere you will be amply repaid for having credited the animal with a certain amount of reasoning power, and you will be astonished at how much he can learn. Some men expect the horse to know more than they do themselves, and are very angry when the poor beast fails to know, by intuition, what is wanted of him, or to get the clue from their rough and barbarous attempts to show him.

In the practice of your profession you will ever find abundant opportunities for the practical application of the results of your studies in comparative psychology. There are a great many difficulties to be overcome for the veterinary surgeon first, and very important is it to acquire the power of so controlling or quieting his patients so that a careful examination may be made and medicines administered or means taken to relieve them. I am not now referring to those means of forcible restraint that frequently have to be made use of. but to what may properly be called moral suasion. You all must, if you have observed the matter at all, have noticed how differently dogs and horses behave with some men than with others. Perhaps you have never looked closely into this question, nor satisfied yourselves as to the reason why. If you do you will find that one approaches the animal in such a way as to disarm all his suspicions ; comes as one friend to another, secure in the anticipation of a friendly reception; the other as if he was frightened himself or had a grievance that he was going to settle at once Animals are very quiet observers, and soon detect, from the style of approach, the spirit of the visitor Most animals have more or less of resentment in their composition, and the fearful one may be met by a threatening attitude. The aggressive one rouses the animosity of the animal, and his manner may be roughly resented. The quiet, confident, conciliatory advance is almost invariably met with the proper reciprocal spirit. The friendly advance is received in a friendly manner, and a favorable, mutual acquaintance is begun. Few animals are naturally vicious, many are constitutionally timid ; fear very soon gives place to a feeling of resentment, and the animal uses his natural means of defense against an unwelcome intruder.

Treat your patients as if they were beings possessed of reasoning powers, and in the great majority of cases your confidence will not be misplaced. It is astonishing how often a barking, snapping dog, or an apparently vicious horse, will yield to friendly manifestations, and will allow almost any liberty to be taken with him. This is not a mere theory, but an actual fact of which I have had innumerable examples. If this is instinct, there is more in instinct than I have hitherto thought.

I have endeavored, in a rambling manner, to bring before you some food for thought, some incentives to pursue your investigations, and have tried to point out some practical applications of the results you may obtain. The student of comparative psychology should have his affections and sympathies warmed toward the lower animals. He should learn to know them better and, as he does so, he should, by realizing their powers and capabilities, and at the same time their dependance on man for not only the further development of those powers, but for the full enjoyment of such powers as he now possesses; and, also, we may say, in his state of subjection to man the power, or rather the means of any enjoyment.

We should ever strive to make the lot of our dumb friends the better, as by having studied them we realize how they can suffer and enjoy. Not merely because of what they do for us do they deserve it, but because we take a pleasure in ministering to their comfort and happiness. The higher the opinion we form of the intelligence of the lower animals, the better will their lot become, and the better they will serve us, for if we give them credit for possessing a certain degree of reasoning power, and there is no doubt but that they do, we naturally treat them better, make their surroundings more pleasant and agreeable, take more pains in making our wishes known, treat them reasonably and are treated in like manner. What we all require is to give to the animals around us a little more thoughtful consideration ; make them our trained friends and not merely unwilling slaves; but never let your kindness go too far in the way of indulgence ; temper your kindness with firmness and vice versa.

